# Validating Deep Neural Networks for Online Decoding of Motor Imagery Movements from EEG Signals

**DOI:** 10.3390/s19010210

**Published:** 2019-01-08

**Authors:** Zied Tayeb, Juri Fedjaev, Nejla Ghaboosi, Christoph Richter, Lukas Everding, Xingwei Qu, Yingyu Wu, Gordon Cheng, Jörg Conradt

**Affiliations:** 1 Institute for Cognitive Systems, Technical University of Munich, 80333 Munich, Germany; gordon@tum.de; 2 Neuroscientific System Theory, Department of Electrical and Computer Engineering, Technical University of Munich, 80333 Munich, Germany; j.fedjaev@gmail.com (J.F.); c.richter@tum.de (C.R.); lukas.everding@tum.de (L.E.); ga62gis@mytum.de (X.Q.); ga62guz@mytum.de (Y.W.); conradt@tum.de (J.C.); 3 Research and Development, Integrated Research, Sydney 2060, Australia; nejla.ghaboosi@gmail.com

**Keywords:** Brain-Computer Interfaces, spectrogram-based convolutional neural network model (pCNN), Deep Learning, electroencephalography (EEG), long short-term memory (LSTM), recurrent convolutional neural network (RCNN)

## Abstract

Non-invasive, electroencephalography (EEG)-based brain-computer interfaces (BCIs) on motor imagery movements translate the subject’s motor intention into control signals through classifying the EEG patterns caused by different imagination tasks, e.g., hand movements. This type of BCI has been widely studied and used as an alternative mode of communication and environmental control for disabled patients, such as those suffering from a brainstem stroke or a spinal cord injury (SCI). Notwithstanding the success of traditional machine learning methods in classifying EEG signals, these methods still rely on hand-crafted features. The extraction of such features is a difficult task due to the high non-stationarity of EEG signals, which is a major cause by the stagnating progress in classification performance. Remarkable advances in deep learning methods allow end-to-end learning without any feature engineering, which could benefit BCI motor imagery applications. We developed three deep learning models: (1) A long short-term memory (LSTM); (2) a spectrogram-based convolutional neural network model (CNN); and (3) a recurrent convolutional neural network (RCNN), for decoding motor imagery movements directly from raw EEG signals without (any manual) feature engineering. Results were evaluated on our own publicly available, EEG data collected from 20 subjects and on an existing dataset known as 2b EEG dataset from “BCI Competition IV”. Overall, better classification performance was achieved with deep learning models compared to state-of-the art machine learning techniques, which could chart a route ahead for developing new robust techniques for EEG signal decoding. We underpin this point by demonstrating the successful real-time control of a robotic arm using our CNN based BCI.

## 1. Introduction

Non-invasive brain-computer interfaces (BCIs) are intelligent systems that enables users to communicate with external devices such as computers or neural prostheses without the involvement of peripheral nerves and muscles. Therefore, BCIs can be applied to a wide range of applications in order to support people with motor disabilities by interacting with their surroundings. BCI-based motor imagery (MI) describes a mental process in which a person solely imagines to perform a certain action, e.g., opening or closing the left or right hand without executing it. Recent research has shown that this type of BCI could allow both healthy and severely paralyzed people to control a robotic arm [1] or to move around in a wheelchair [2]. Notwithstanding the good results obtained by previous work focusing on the well-known MI patterns [3], the progress in BCI performance has been stagnating in the last decade. Apparently, the design of more stable classification methods is comprising formidable challenges. One of these challenges stems from the low signal-to-noise ratio (SNR) of electroencephalography (EEG) signals as well as the high variability in recordings across trials and within participants, which puts the identification of more robust and discriminative features in EEG-based BCI systems under high demand. This work focuses on the decoding of two MIs, namely left-and right-hand movements. It shows the potential of using deep learning methods to classify binary MI movements in EEG signals. Three different deep learning models have been investigated and tested. These models are LSTMs [4], CNNs [5], and RCNNs which have until now been rarely investigated for BCI applications [6,7,8,9]. In addition, more than six different machine learning classifiers have been implemented and used for the purpose of comparison with the aforementioned deep learning methods. The remainder of this paper is organized as follows: Section 2 presents an overview of related work and reviews scientific research on classifying MI movements using deep neural networks (DNN). Section 3 describes the implementation details of the different proposed deep learning models. Section 4 shows the obtained results using deep neural networks as well as traditional machine learning methods on our recorded EEG data and the publicly available dataset 2b from BCI Competition IV [10]. Real-time control of a robotic arm using our trained CNN model on EEG signals is described in Section 5. Finally, Section 6 enumerates the strengths and weaknesses of our proposed methods and proposes possible future improvements.

## 2. Related Work

This section provides a review of different deep learning approaches developed for EEG decoding.

### 2.1. Recurrent Neural Networks (RNN)

#### 2.1.1. Elman Neural Network

Elman Neural Network (ENN) is a type of RNN which was proposed by Jeff Elman. In ENN with multiple stacked hidden layers, each hidden layer receives the activation of previous hidden layers per timestep. The feedback connection enables the neural network to capture the temporal patterns, which makes RNN a huge success in natural language and video processing. In [11], an Elman network was used to classify EEG data during a 3-D perception task where subjects were viewing either 2D or 3D images. In that paper, the authors showed that an accuracy of 58% can be achieved using ENN whereas, an accuracy of 72% was obtained on the same task using a two layer multilayer perceptron (MLP). Due to the unsatisfactory obtained performance, the authors concluded that it is not straightforward to apply RNNs to EEG data. In [12], the authors used an ENN to classify MI mental tasks such as right hand clenching, observing a tumbling cube and silently singing a song. Three subjects participated in the study and the accuracy varied drastically across subjects from 58.8% to 93.3% reflecting a large intra-subject variability. In [13], authors used only two electrodes, namely, C3 and C4, and achieved 93% accuracy for four MI movements classification using an ENN.

#### 2.1.2. Long-Short Term Memory (LSTM)

Since ENNs share the same weight matrix at different time steps, this can cause either the gradient exploding or the gradient vanishing problem. To address that, Sepp Hochreiter and Jürgen Schmidhuber proposed the LSTM network in 1997 [4], where simple neurons are replaced by LSTM units each consisting of four main components: an input gate, a neuron with a self-recurrent connection, a forget gate and an output gate [4]. In [14], with the use of a large EEG dataset (109 subjects, 26.4 million samples) from PhysioNet eegmmidb [15], an accuracy of 95.53% was achieved for five MI movements classification problem using seven RNN layers with two LSTM layers. It is worth noting that unlike [12], the results obtained using PhysioNet dataset were stable over all subjects. However, the developed model was only tested with the large scale PhysioNet dataset and no evaluation on their own recorded dataset was performed. In [16], five different recurrent neural network architectures were tested for hand motion recognition of grasp-and-lift task from EEG signals, namely LSTM, GRU, MUT1, MUT2, and MUT3 [17]. The obtained results showed that MUT3 performs the best with an accuracy of 88.82%, whilst LSTM and GRU had an accuracy of 87.89% and 88.60%, respectively.

### 2.2. Convolutional Neural Networks (CNN)

Classifying raw EEG data without hand-crafted features is a challenging problem. Schirrmeister et al. [18] pioneered the development of a CNN capable of decoding movement-related information from raw data. Deep as well as shallow-CNNs with various design choices were introduced and compared. The first convolution of the deep-CNN (dCNN) was split into a convolution across both time and space. In the shallow-CNN (sCNN), the first two layers were split into temporal and spatial convolutions. The rest of the networks contained standard convolution-max-pooling blocks with a softmax classification layer at the end. Moreover, the results were evaluated on different frequency bands. By including recent advances from the field of deep learning, such as batch normalization and exponential linear unit activations, the authors obtained accuracies of 71.90% and 70.10% on BCI dataset IVa for dCNN and sCNN models, respectively. We wish to mention that both dCNN and sCNN models have been reimplemented in this work for benchmarking purposes and their architectures are described in the Methods section. In [19], a generalized neural network architecture, called EEGNet, was implemented which is capable of solving different BCI tasks. EEGNet contained three layers. The first layer learns sixteen 1D convolutional kernels. In the second and third layers, four 2D convolutional kernels are learned and 2-D max-pooling is applied. Furthermore, the model iterates between convolutions along spatial dimension (layer 1 and 3) and temporal dimension (layer 2). In each layer, the Exponential Linear Unit activation function is used and Batch Normalization and Dropout are applied to improve the model robustness. An overall accuracy of 70% was achieved using EEGNet on four MI movements.

### 2.3. Recurrent Convolutional Neural Networks (RCNN)

In [20], the authors proposed to construct the spectral information over the whole trial duration as a sequence of images, and used RCNN, which is a combination of RNN and CNN, for four-classes working memory task classification. The obtained results showed that deep RCNN is capable of learning robust representations from sequence of images. They also demonstrated that their proposed model outperforms the state-of-the-art traditional machine learning approaches. In addition, in [21], authors proposed an RCNN model for six-class hand motion classification problem with 94.8% accuracy. They also showed that their RCNN architecture outperforms CNN. It should be noted that this high accuracy was only obtained for hand motion tasks and was not tested with motor imagery movements.

## 3. Methods

### 3.1. EEG Signals Recording

**Figure 1:**
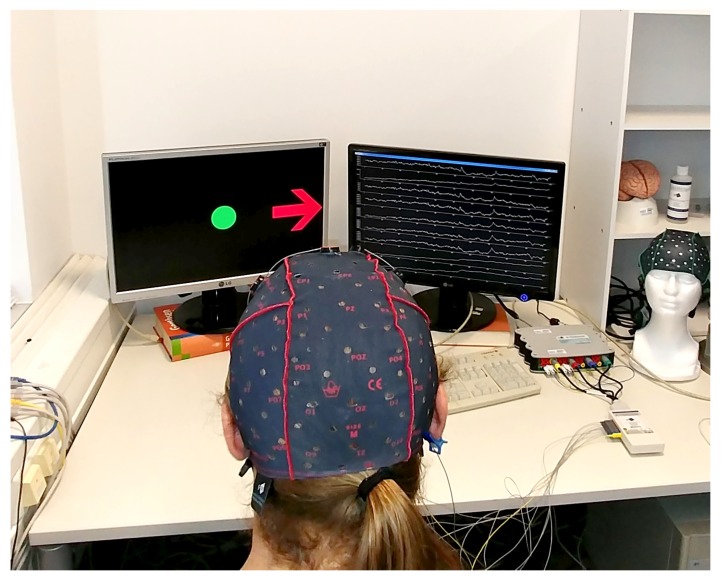
Experimental setup and an example of a recording session of motor imagery-electroencephalography (MI-EEG) recording.

For MI-EEG data recording, 20 healthy and right-handed human subjects (31±5.5 years old) were recruited to perform a series of kinesthetic MI tasks across 2 sessions yielding a total of 750 trials. All recording sessions took place at our lab and were set up as shown in Figure 1. Each session consisted of 4 runs of 12 min separated by 10-min breaks to avoid mental fatigue. Each run consisted of several MI tasks, each 10 s long. At t=4.5s, an arrow pointing either to the left or right was displayed with an acoustic warning tone (1 kHz, 70 ms). The subject was instructed to imagine a movement according to the displayed cue (left vs. right) for four seconds. The MI task was followed by a relaxation period of 1.5 s that separated two trials. During the recording, no movement execution was requested. Data were recorded and sampled at 256 Hz using a g.tec g.USBamp EEG system [22] with 32 active electrodes located according to the 10/20-system. The number of electrodes was reduced to three, namely C3, C4, and Cz, over the sensorimotor cortex. The experimental paradigm is made publicly available with the gumpy toolbox [23]. 375 trials were recorded for each MI movement (left and right) with every subject.

### 3.2. Data Preprocessing

EEG signals were processed using the gumpy.signal module in the gumpy BCI toolbox [23]. First, a notch filter at 50 Hz was applied in order to remove power line interference. Second, data were high-pass filtered with a cutoff frequency of 0.5 Hz to remove baseline drift and then band-pass filtered between 2 and 60 Hz using a 5th order zero-phase Butterworth filter. Afterwards, the EEG data were clipped to $\mu \left(x_{i}\right)\pm \;6\sigma \left(x_{i}\right)$ rectifying outliers. $\mu (x_{i})$ and $\sigma (x_{i})$ denote, respectively, the mean and standard deviation for the EEG data of channel *i*. Next, the data were normalized by subtracting $\mu (x_{i})$ from each channel *i* and then dividing by the standard deviation $\sigma (x_{i})$. Furthermore, a thresholding-based method [24] to detect and remove EOG and EMG artifacts from EEG was used. For that, we removed artifacts based on the mean amplitude value and standard deviation from individual channels within single epochs. Overall, deleted portions and epochs from the data were generally characterized by high amplitude values >83μV.

Finally, in order to reflect the partial time invariance of the data and overcome the problem of overfitting, a data augmentation method was performed: A time window of 4s was used to create different crops with a stride of 125ms yielding 25 new sub-trials from each individual trial. The crops were gathered starting 3 s prior to the motor imagery onset until the end of the trial. Noticeably, this augmentation method was very helpful and forced our proposed “pragmatic” CNN model (see Section 3.3.2) to learn complex features from all the crops and therefore led to better classification performance. In total, this cropping strategy increases the training set by a factor of 25 yielding 25 new examples per trial and a total number of 18,750 trials (9375 for each class).

### 3.3. Decoding Methods

A BCI’s decoding stage aims to extract usually distinct commands—the user intention—from the complex, multidimensional EEG data stream. Different strategies and myriad methods could be applied to this problem, which is essentially a classification task. In this work we systematically compare traditional ML methods to contemporary neural ones. Within the scope of this work the categorical difference between the traditional and the neural approach is that only the latter can perform well on raw EEG data (or a direct representation thereof), whereas the former would typically rely on “hand-crafted” features. These are discriminative data properties like, e.g., the power in specific frequency bands at specific recording electrodes. They are specific to the individual task and experimental paradigm and are commonly defined and selected by an expert—a task we term “feature hand-crafting” or “feature engineering”. The specificity and discriminative power of an individual feature would often vary among different subjects and different trials. Automatic (optimizing) feature selectors can only partly counteract this tendency.

Certain neural networks, in contrast, can classify EEG data directly: RNNs can evaluate EEG time series [11]; CNNs can classify [19] (series of) spectrograms. The networks can apparently find discriminative features automatically, without user intervention, without hand-crafted features. This is beneficial, because (1) less “hand-crafting” is required to define task-specific features, (2) potentially more complex spatio-temporal features can be found, and (3) the process of selecting or weighting relevant features is more dynamic and adaptive, so inter-subject and inter-trial variability should eventually be reduced.

The remainder of this section details the employed neural networks, traditional ML classifiers, and features.

#### 3.3.1. LSTM Model

Over the last few years, recurrent neural networks, mainly LSTM has gained tremendous momentum and prevalence for a variety of applications such as sequence to sequence generation and time-series prediction. As LSTM models are capable of learning long-term dependencies in time-series data, it would be appropriate to investigate their potential in classifying MI from EEG data. Hence, we developed an LSTM network with one hidden layer containing 128 cell units followed by an additional fully-connected layer that consists of two output neurons representing the two classes “left and right hand movements”. In regard to the model’s architecture choice, it is worth mentioning that using more layers and memory cells improved the training accuracy, but led to overfitting during the test phase, due to the exponential number of parameters. Likewise, reducing the number of memory cells to less than 128 led to underfitting shown by a significant decrease in both training and validation accuracies. Moreover, a dropout layer with a deactivation rate of 0.05 was used between the LSTM layer and the output to alleviate overfitting. The network was trained using a stochastic gradient descent on mini-batches of size 256 and using categorical cross-entropy as the loss function on a NVIDIA GTX Titan X GPU, with CUDA 8.0 and cuDNN v5, using Theano 0.9 [25] and Keras library [26]. For each of the 20 participants, training has been conducted using a stratified 5-fold cross-validation. More precisely, one of the five folds was held back for testing (3750 trials) while the four remaining folds (15,000 trials) were used for training and validation with a split of 90% and 10%, respectively, in a loop until each fold has once been used for testing. Stratified in this context means that both classes are represented equally in each fold.Finally, we point out that early stopping [27] was used to avoid overfitting. That means the model is trained until the minimum of the validation loss is found and then tested on the test data split to measure its generalization capabilities.

#### 3.3.2. The Pragmatic CNN Model (pCNN)

In addition to reimplementing the two aforementioned CNN models (shallow sCNN and deep dCNN) by Schirrmeister et al. [18] we have developed a third CNN which we term “the pragmatic CNN” (pCNN). In terms of complexity the pCNN is in between of sCNN and dCNN. As will be shown the model can classify MI tasks with a high accuracy, yet it is sufficiently light-weight to perform real-time control of a robotic arm.

**Figure 2:**
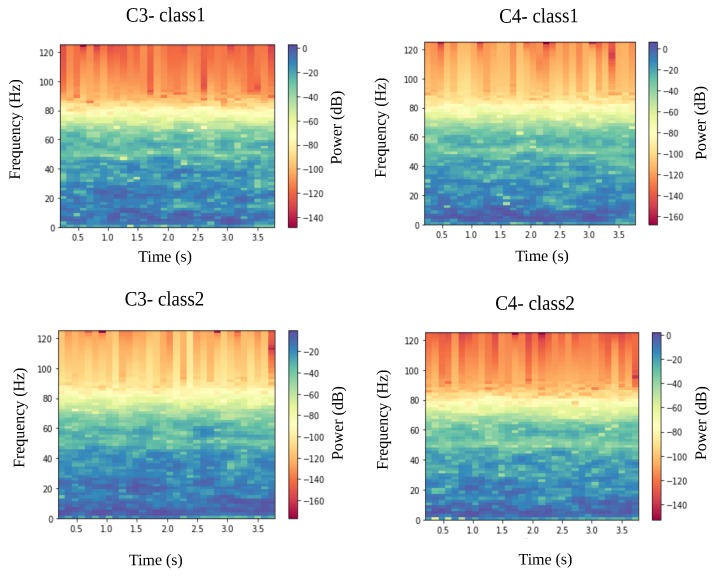
Example of the generated spectrograms from C3 and C4 electrode during left (class 1) and right (class 2) hand movements imagination.

Given that EEG data have a time-series structure, it was paramount to convert them into an image-like representation by computing spectrograms using a short-time Fourier transform (STFT), which is a well-known technique in audio signal processing [28]. An example of the obtained spectrograms during left and right imagined movements is shown in Figure 2. Overall there is a subtle, but clear difference in the generated spectrograms between the two imaginations on the same electrode in the frequency range of 25–50 Hz. However, it remains challenging to recognize the event-related (de)synchronization (ERD) in the alpha band between 8–13 Hz and lower beta band (14–24 Hz) [29] from the generated spectrograms.

**Figure 3:**
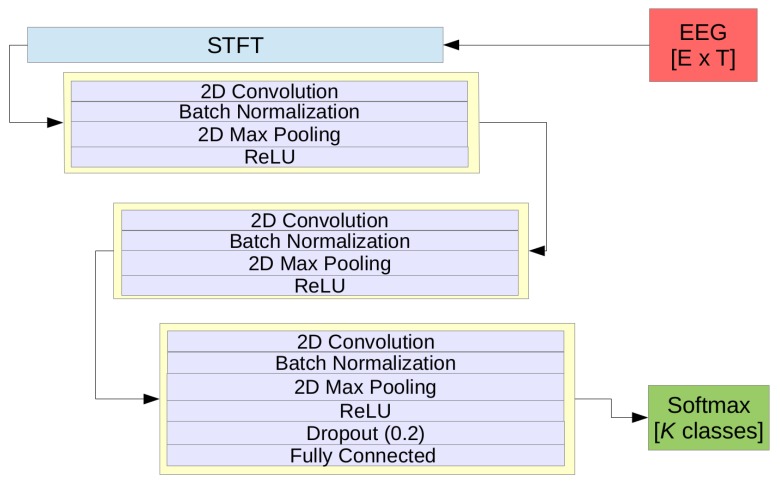
The pragmatic conventional neural network (pCNN) model’s architecture, where *E* is the number of electrodes, *T* is the number of timesteps, and *K* is the number of classes.

**Figure 4:**
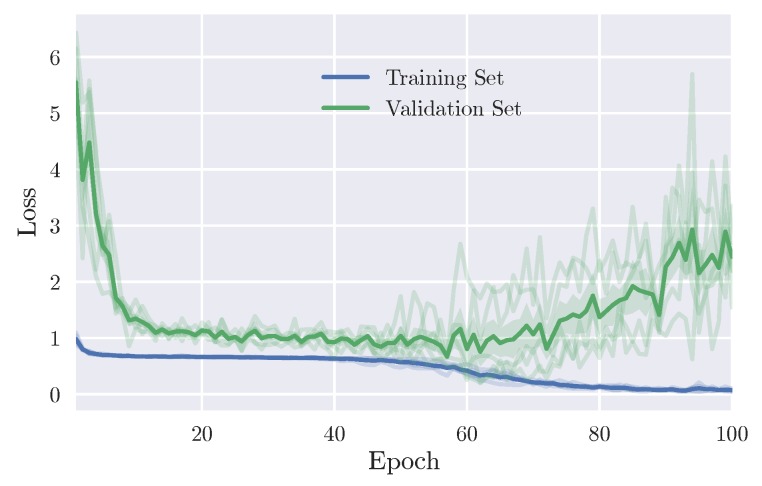
Training and validation loss of the pCNN model. The blue and green lines represent the average of the 5 folds for training and validation, respectively.

Computed spectrograms in the input layer are fed into our pragmatic CNN model (pCNN), which contains three convolutional blocks. Each block contains one convolutional layer, batch normalization to minimize covariate shift and enhance the robustness of the model [30], and max-pooling layer with a downsampling factor of 2 between each layer. A rectified linear unit (ReLU) is used as the activation function. Finally, a fully connected layer with a softmax activation function is used to compute the probability of each class. Weights were learned using the Adam optimizer [31]. The network architecture is shown in Figure 3. Similar to LSTM, the CNN network was trained using the same procedure. Overall, Figure 4 shows the fast convergence of the CNN model. It should be noted that the early stopping strategy was used to efficiently find the best model. The full architecture of the pCNN model can be found in Table A1 in the Appendix.

As shown in Figure 4, at epoch 62 the validation loss starts increasing as opposed to the continued decrease of the training loss. This indicates the overfitting problem which could be explained by the small amount of data that is used for training. Hence, the early stopping technique, as aforementioned, was chosen in order to save the best model.

#### 3.3.3. RCNN Model

As was shown in Figure 2 above, it remains unclear whether the computed spectrograms encode enough information from EEG signals and whether the CNN could learn high-level features from them. Additionally, it may be desirable to improve the CNN model’s ability to integrate the context information, which might be of utmost importance while learning the sequence of spectrograms in EEG signals. Consequently, an RCNN model has been implemented and its capability of learning MI movements has been investigated. The key module of such a model is the recurrent convolutional layer (RCL) [32], which can be seen as a specific form of RNN. In the RCL, the feed-forward and recurrent computation both take the form of convolution. During the training and test phase, the RCL is unfolded through discrete time steps into a feedforward subnetwork. The number of time steps, namely recurrent iterations, is pre-fixed as a hyper-parameter. Overall, RCNN can be described as a stack of these RCLs. The proposed RCNN architecture is described in Table 1.

#### 3.3.4. Shallow-CNN (sCNN) and deep-CNN (dCNN)

To benchmark our obtained results we validated with three aforementioned models (LSTM, pCNN, RCNN), and two deep learning models (dCNN, sCNN) proposed recently by Schirrmeister et al. [18]. The dCNN model consists of four convolutional-max-pooling blocks followed by a dense softmax classification layer. Unlike previous implementations, the first convolution block is split into two parts where filters of the first one learn temporal information and the ones of the second layer learn 2D spatial information from the already learned temporal layers. On the other hand, the sCNN architecture which is inspired by the filter bank common spatial patterns (FBCSP) [33] relies essentially on band power features. For more technical details about the models’ architecture, readers are referred to [18]. The full architecture of the dCNN and sCNN models can be found in Table A2 and Table A3, respectively, in the Appendix.

#### 3.3.5. Traditional Machine Learning Approaches

Aside from the deep learning techniques, we implemented and tested a range of classical machine learning approaches which are based on hand-crafted features. Five different classifiers from the gumpy.classification module [23] have been used and evaluated in order to provide a baseline for the deep learning models: *K*-Nearest Neighbor (KNN), Decision Tree (DT), Logistic Regression (LR), Naive Bayes (NB), and Quadratic Linear Discrimination Analysis (QLDA). Three different feature extraction methods were used, namely logarithmic band power (log-BP) [34], common spatial patterns (CSP) [35] and discrete wavelet transform [36]. For the log-BP method, we analyzed the log-power of mu rhythm (8–12 Hz) and beta (14–30 Hz). For each of the three channels (C3, C4, and Cz), log-BP features in 72 frequency bands were calculated using different overlapping narrow bands between 8 and 30 Hz yielding a total of 216 BP features. Additionally, the CSP method, which maximizes the pairwise compound variance between our two classes in the least square sense, was implemented and used for benchmarking purposes. Furthermore, statistical features (mean, root mean square (RMS) and standard deviation (SD)) were extracted from D3 and D4 wavelet coefficients. Thereafter, a feature selection algorithm [37] was used for each feature extraction method to select a subset of features. A 5-fold cross validation was performed and features were fed into the classifiers in order to discriminate between the two classes.

## 4. Results

Our motor imagery data recorded from 20 subjects were used to compare the models’ performance. In order to further verify our results on independent data, we additionally used Graz dataset 2b from BCI Competition IV [6]. It should be noted that balanced accuracy was chosen as the evaluation metric for the trained models and a stratified 5-fold cross-validation was applied during the validation phase.

### 4.1. Results on Our EEG Recorded Dataset

#### 4.1.1. Traditional, Baseline Classifiers

**Figure 5:**
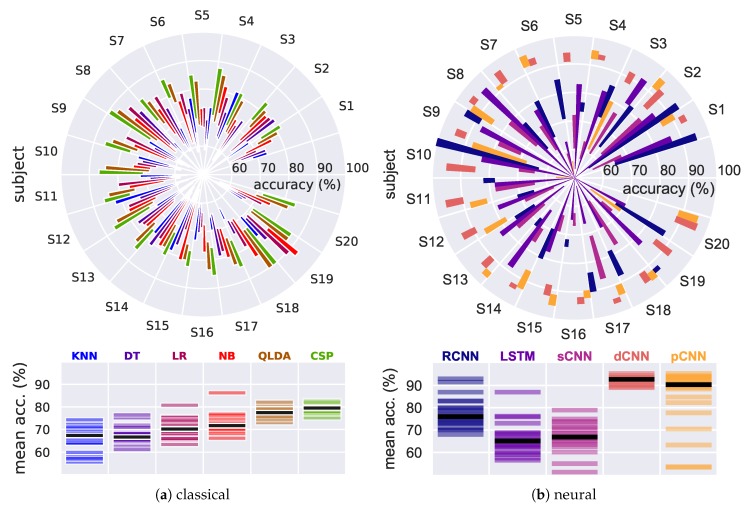
MI classification accuracies from 20 subjects using (**a**) traditional machine learning approaches and (**b**) different neural classifiers. The polar bar plot shows the accuracy range (mean ± standard deviation) achieved by the 5 models for each of the 20 subjects. The lower panel subsumes for each algorithm the 20 mean accuracies achieved, black bars indicate the median result.

Figure 5a presents the results of the traditional classification algorithms. Overall, QLDA outperforms all the other classifiers with a mean accuracy over all subjects of 79.5% with CSP features and 78% with log-BP features. DT performs the worst with a mean accuracy of 67%. According to their performance with the QLDA classifier, the 20 participants could be classified into three groups: (G1) Participants S3 and S14 achieved a mean accuracy below 75%. (G2) Participants S1, S2, S4, S5, S7, S8, S9, S10, S11, S12, S13, S15, S16, S17, S19, and S20 achieved a mean accuracy between 75% to 79%. (G3) Participants S6 and S18 reached a mean accuracy of 80.52% and 82.09%, respectively. It should be noted that an average mean accuracy of 75% was obtained using the wavelet method when tested with QLDA.

#### 4.1.2. Neural Models

Figure 5b compares the classification accuracies achieved using the developed neural classifiers (RCNN, LSTM, pCNN) and two other models dCNN and sCNN proposed by Schirrmeister et al. [18]. It is worth noting that the dCNN and pCNN models outperformed all the other developed classifiers and attained higher accuracy.

##### LSTM Model

To assess the LSTM’s capability of learning discriminative features, only raw EEG signals were fed into the model. Noticeably, obtained results show a high standard deviation (SD) within subjects and across the test splits. Overall, a mean accuracy of 66.2% (±7.21%) from all subjects was achieved for two classes. Interestingly, the LSTM model could perform well with some of the subjects e.g., S3 with a reached accuracy of 86.97% (±5.18%). Contrary, an average accuracy of only 60% was obtained from S6, S16, S18, and S20 which could be due to the fact that the raw data collected from these subjects were too noisy and hence the LSTM model could not learn any discriminative features. As illustrated in Figure 5b, the LSTM-based raw EEG data approach did not outperform any of the other developed models and the results remained slightly inferior to those obtained by state-of-the-art methods as will be shown in the next sections.

##### CNN Models

Herein, we show the obtained results using our pCNN and two other CNN models proposed by Schirrmeister et al. [18] which have been reimplemented in this work for benchmarking purposes. Through all subjects, a mean accuracy of 84.24% (±14.69%) was achieved using the pCNN model. We wish to emphasize that such a high standard deviation could be easily explained by the failure of the model to classify recorded EEG signals from S5, S10, and S19 as shown in Figure 5b. Although reasons for that remain unclear, we could explain that some of these participants were unable to properly imagine the requested MI movements which resulted in a low signal to noise ratio of the collected data, and hence most of the developed deep learning models (except the dCNN model) failed to classify them. Overall, it is important to highlight that the pragmatic model (pCNN) shows a high stability across test splits with a mean SD of less than 3.32%.

Furthermore, the achieved results with the sCNN model are also shown in Figure 5b The mean accuracy over all the participants is 66.97% (±6.45%), which is barely lower than the presented results in [18] and close to the one obtained with the LSTM model. However, it should be noted that the sCNN shows less variance in the obtained accuracy across subjects and within different data splits compared to LSTM.

Finally, a mean accuracy of 92.28% (±1.69%) was obtained with the dCNN model, which is better than the pCNN model. However, it should be noted that the dCNN model requires a significant amount of computation due to its extremely large number of parameters. Such a large model requires a large number of floating point operations and can run in a data center. But, for neurorehabilitation devices, the model should be small enough to be fitted into the memory. This makes the pCNN model a better choice as it requires about 50% less memory and computation compared to the dCNN.

##### RCNN Model

A mean accuracy of 77.72% (±6.50%) was obtained with the RCNN model as illustrated in Figure 5b. Overall, RCNN model seems to provide better accuracy than the LSTM and sCNN but worse accuracy than both the pCNN and dCNN. Furthermore, we wish to highlight that the RCNN model required more epochs of training to avoid overfitting.

### 4.2. Results on EEG Graz Dataset

**Figure 6:**
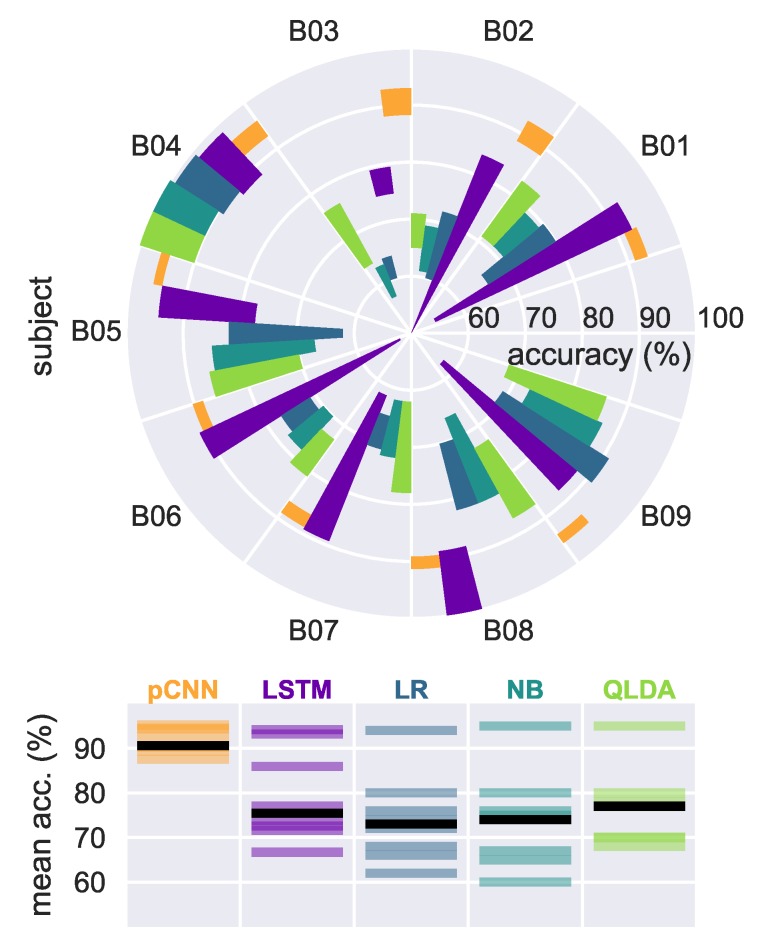
MI classification accuracies from nine subjects using five different classifiers. The polar bar plot shows the accuracy range (mean ± standard deviation) achieved by the five models for each of the nine subjects. The lower panel subsumes for each algorithm the nine mean accuracies achieved, black bars indicate the median result.

To further evaluate the trained models’ performance, we tested the pCNN architecture as well as the LSTM model on the Graz data set B from the BCI Competition 2009 [6]. The data consist of three bipolar recordings (C3, Cz, and C4) sampled at 250 Hz and two classes, namely the MI of left and right hand. Figure 6 shows that the pCNN model outperforms both LSTM, Naive Bayes and quadratic LDA for all nine subjects except for subject B08. For the LSTM model, better results than Naive Bayes and quadratic LDA are obtained with subjects B03, B05, B07, B08 wheareas quadratic LDA with the log-power features provided better mean accuracy results for subjects B01, B02, B04 B06, B09. Overall, models’ performances are aligned with previously obtained performance on our recorded EEG data with lower variance for the pCNN model. We wish to mention that a mean accuracy of 95.72% and 78.22% was obtained through all subjects, using the dCNN and sCNN, whereas a mean accuracy of 91.63% and 78.93% was achieved using the pCNN and the LSTM models, respectively.

## 5. Real-Time Control of a Robot Arm

**Figure 7:**
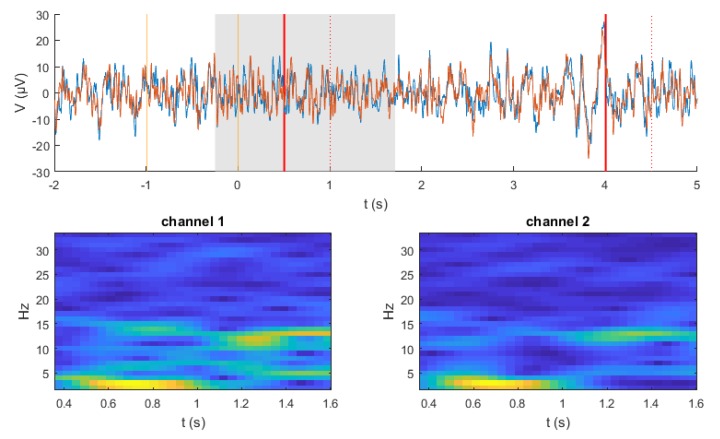
A frame of a live stream. **Top**: Filtered signal during a trial. Blue and red traces illustrate channel 1 and channel 2, respectively. Vertical lines indicate visual (orange) and acoustic cues (red). **Bottom**: Generated spectrograms from data within the grey rectangle shown above.

We further tested and validated the real-time capability of the pCNN by online decoding of MI movements from streamed EEG signals for a robot arm control. Three electrodes were used to record live EEG data. First, a band-pass filter between 2–60 Hz as well as notch filter at 50 Hz were applied. All filters were implemented as Butterworth IIR filters. Second, the experimental setup shown in Figure 1 and the lab streaming layer (LSL) [38] were used to continuously stream the created spectrograms from live EEG data within a circular buffer that stores a predetermined number of samples up to the most recent one. The process of creating spectrograms from EEG streaming data is shown in Figure 7.

**Figure 8:**
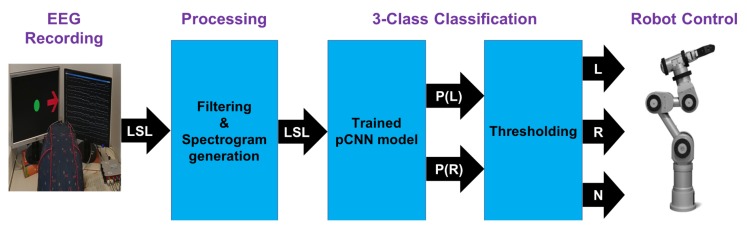
Live setup for real-time EEG signal decoding and Katana robot arm control. P(L) and P(R) represent the probability of left and right hand movements, respectively.

Thereafter, live spectrograms were fed into the trained pCNN model for real-time classification and a robot arm was controlled accordingly. A Katana robotic arm was directed to either move to the left, right or stay in middle position according to the decoded movement from EEG signals. The trained model provides the probability of left and right hand movements. To detect the stay position, we defined a threshold for the classifier. That means signals with a high probability to move (left or right movements) are classified accordingly, and the remaining are categorized as no movements. The whole process is depicted in Figure 8. A video of a successful live demo is available in the Supplementary Materials Section. The live generation system using the trained pCNN has been tested for frame rates up to 128 Hz on a PC with a 2.8 GHz quadcore CPU. As shown in the Supplementary Video, a noticeable delay (∼1.4 s) was experienced when performing the real-time experiment using the pCNN model. This delay can be attributed almost entirely to the CNN processing. A delay of ∼2.55 s is expected when using the trained dCNN model.

## 6. Discussion

In this paper, three different deep learning models were proposed to classify MI movements from our recorded EEG signals: a time-series based LSTM, a pCNN, and a RCNN model. In addition, the deep- and shallow-CNN models proposed in the literature [18] were reimplemented for benchmarking purposes. Furthermore, five classic machine learning classifiers were also used for comparison. Several models for BCI-based MI have been proposed in the past. However, the present work is one among very few to show an online decoding of motor imagery movements using the pCNN model. Overall, with a classification accuracy of (66.2±7.21)% the LSTM model performed similar to the sCNN model with (66.97±6.45)% accuracy. Despite the LSTM’s capability of learning time-series sequences, the LSTM model did not outperform our pCNN, the dCNN, or even well-known machine learning classifiers that are commonly used in BCI such as SVM and quadratic LDA. As LSTM is prone to overfitting, one intuitive reason for the inferior results (with respect to [14]) could be the limited amount of training data which at the same time hindered the development of more complex LSTM models with more layers and cell units. On the other hand, our pCNN showed better performance compared to the LSTM model. The obtained performance could be due to the capability of CNNs to learn complex nonlinear features as well as the fact that the time-frequency representation of the signal was used as an input to the model. Also, interestingly, the pCNN model showed higher stability in both training and validation accuracy compared to the other models despite the existence of overfitting potential due to its high number of parameters (170,734). The dCNN model showed promising results and slightly outperformed all the trained models confirming the ability of convolutional models in general to extract complex and discriminative features. Moreover, it confirmed that deeper networks could provide more accurate results. However, it should be noted that the large depth of very deep models like dCNN could result in an extremely high number of parameters (268,977), which in turn causes the model to be computationally expensive. As a “pragmatic” alternative, our pCNN provides a much better computation-accuracy trade-off and hence makes it a more attractive choice for the type of applications that we are interested in. It is also worth mentioning that unlike previous obtained results in [18], the performance of the shallow model was vastly inferior to the dCNN (66.97% vs. 92.28%). Although the reasons for that remain unclear, we could explain this inferiority by a poor choice of the optimizer’s hyper-parameters. These hyper-parameters have to be carefully chosen for models like sCNN and dCNN as they do not rely on standard activation functions such as ReLu, exponential ReLu, sigmoid, and Tanh.

Lastly, the RCNN model showed better performance than the developed LSTM, sCNN, and classic machine learning methods with a mean accuracy of (77.72±6.50)%, but not the dCNN and pCNN models. However, RCNN could be subject to further improvement by optimizing the network architecture. Thus, a different RCNN architecture as presented by Bashivan et al. [20], that combines CNN and LSTM, could improve the achieved results. Finally, it should be noted that the amount of data used to train all of the aforementioned models were limited. Therefore, transfer learning could be applied in future work to pre-train our developed models on larger datasets and use the acquired knowledge thereafter to classify more complex movements from our recorded EEG data, such as reach-to-grasp movements.

## 7. Conclusions

This paper thoroughly describes the details of three deep learning models (LSTM, pCNN, RCNN) for online decoding of imagined hand movements from EEG signals. Additionally, the developed DNN models were compared with two other models (dCNN, sCNN) proposed in the literature. Overall, the two CNN architectures (dCNN, pCNN) showed better performance and achieved a mean accuracy higher than 84% over all the 20 participants, the RCNN model reached a mean accuracy of 77.72% and a comparable accuracy to state-of-the-art results was obtained with the LSTM model. In general, the traditional way of decoding EEG data has consisted in (1) data preprocessing, (2) feature engineering, and (3) classification. Methods for all three processing steps abound, but have to be carefully selected and linked together by experts. Neural networks can merge steps (2) and (3), and identify relevant data features automatically. Their crucial benefit is not that they require less expert knowledge, rather it is that they can automatically and dynamically adapt their selection and weighting of features to different trials, different subjects, and possibly also different tasks [39]. Our data confirm at least the two former points: Sufficiently deep convolutional neural networks achieve less inter-trial and less inter-subject variability. Overall they classify significantly more accurately than traditional methods. This is, however, quickly becoming easier and more accessible thanks to powerful and user-friendly software frameworks like Keras [26] or EEG (and EMG) specific toolkits like gumpy [23]. Furthermore, a successful real-time control of a robot arm was achieved using the trained pCNN model. The live demo accompanying this paper was run on an x86 CPU, which resulted in a 1 s delay when executing our pCNN. Using state-of-the-art neural processing units (NPUs) [40], this setup can be enhanced to be (1) faster, (2) power saving, and (3) much smaller, all at the same time. So it appears very likely that NPUs, not CPUs will power future generations of neurorehabilitation devices, such as exoskeletons and neuroprostheses. IBM’s TrueNorth neuromorphic hardware [41] could be one attractive target platform: With a power consumption of less than 70 mW and a compact size, IBM’s chip could present an ideal platform for neurorehabilitation devices.

## Figures and Tables

**Table 1:** The proposed recurrent convolutional neural network (RCNN) architecture

Layer Type	Size	Output Shape
Convolutional	256×9 filters	(144,1,1280,256)
Max pooling	Pool size 4, stride 4	(144,1,1280,256)
RCL	256 filters (1×1), 256 filters (1×9), three iterations	(144,1,1280,256)
Max pooling	Pool size 4, stride 4	(144,1,320,256)
RCL	256 filters (1×1), 256 filters (1×9), three iterations	(144,1,320,256)
Max pooling	Pool size 4, stride 4	(144,1,80,256)
RCL	256 filters (1×1), 256 filters (1×9), three iterations	(144,1,20,256)
Max pooling	Pool size 4, stride 4	(144,1,5,256)
RCL	256 filters (1×1), 256 filters (1×9), three iterations	(144,1,20,256)
Max pooling	Pool size 4, stride 4	(144,1,5,256)
Fully connected	1280×3	(144,2)

## Supplementary Materials

Dataset: http://gumpy.org/. The source code of the different developed deep learning models is available with a detailed documentation at https://github.com/gumpy-bci/gumpy-deeplearning. In addition, we provide a tutorial-like overview in the form of Jupyter notebook examples. A Supplementary Video is available here: https://www.youtube.com/watch?v=8hM7tOd7M7A.

## Appendix A

**Table A1:** Pragmatic CNN (pCNN) architecture. *E* is the number of channels, *T* is the number of timesteps and *K* is the number of classes. Input and Output sizes are shown for cropped training with E=3 (electrodes C3, C4, and Cz) and T=1024 for window size of 4 seconds; binary classification with two classes for K=2.

Layer	Input	Operation	Output	Parameters
1	E×T	STFT	65×64×E	-
2	65×64×E	24×Conv2D(12×12)	65×64×24	10,392
	65×64×24	BatchNorm	65×64×24	260
	65×64×24	MaxPool2D (2×2)	32×32×24	-
	32×32×24	ReLU	32×32×24	-
2	32×32×24	48×Conv2D(8×8)	32×32×48	73,776
	32×32×48	BatchNorm	32×32×48	128
	32×32×48	MaxPool2D (2×2)	16×16×48	-
	16×16×48	ReLU	16×16×48	-
3	16×16×48	96×Conv2D(4×4)	16×16×96	73,824
	16×16×96	BatchNorm	16×16×96	64
	16×16×96	MaxPool2D (2×2)	8×8×96	-
	8×8×96	ReLU	8×8×96	-
	8×8×96	Dropout (0.2)	8×8×96	-
4	8×8×96	Flatten	6144	-
	6144	Softmax	*K*	12,290
Total				170,734

**Table A2:** Deep CNN (dCNN) architecture as proposed by Schirrmeister et al. [18] (re-implemented in this work), where *E* is the number of channels, *T* is the number of timesteps and *K* is the number of classes. Input and Output sizes are shown for cropped training with E=3 (electrodes C3, C4, and Cz) and T=1024 for window size of 4 s; binary classification with two classes for K=2.

Layer	Input	Operation	Output	Parameters
1	E×T	25×Conv2D (1×10)	E×1015×25	275
2	E×1015×25	Reshape	E×1015×25×1	-
	E×1015×25×1	25×Conv3d (3×1×25)	1×1015×1×25	1900
	1×1015×1×25	BatchNorm	1×1015×1×25	100
	1×1015×1×25	ELU	1×1015×1×25	-
	1×1015×1×25	MaxPool2D (3×1)	338×25×1	-
	338×25×1	Dropout (0.5)	338×25×1	-
3	338×25×1	50×Conv2D (10×25)	329×1×50	12,550
	329×1×50	BatchNorm	329×1×50	200
	329×1×50	ELU	329×1×50	-
	329×1×50	MaxPool2D (3×1)	109×1×50	-
	109×1×50	Dropout (0.5)	109×1×50	-
4	109×1×50	Reshape	109×50×1	-
	109×50×1	100×Conv2D (10×50)	100×1×100	50,100
	100×1×100	BatchNorm	100×1×100	400
	100×1×100	ELU	100×1×100	-
	100×1×100	MaxPool2D	33×1×100	-
	33×1×100	Dropout (0.5)	33×1×100	-
5	33×1×100	Reshape	33×100×1	-
	33×100×1	200×Conv2D (10×100)	24×1×200	200,200
	24×1×200	BatchNorm	24×1×200	800
	24×1×200	ELU	24×1×200	-
	24×1×200	MaxPool2D (3×1)	8×1×200	-
6	8×1×200	Flatten	1600	-
	1600	Softmax	*K*	3202
Total				268,977

**Table A3:** Shallow CNN (sCNN) architecture by Schirrmeister et al. [18] (re-implemented in this work), where *E* is the number of channels, *T* is the number of timesteps and *K* is the number of classes. Input and Output sizes are shown for cropped training with E=3 (electrodes C3, C4, and Cz) and T=1024 for window size of 4 seconds; binary classification with two classes for K=2.

Layer	Input	Operation	Output	Parameters
1	E×T	40×Conv2D (1×25)	E×1000×40	1040
	E×1000×40	Dropout (0.5)	E×1000×40	-
2	E×1000×40	Reshape	E×1000×40×1	-
	E×1000×40×1	40×Conv3D (3×1×40)	1×1000×1×40	4840
	1×1000×1×40	BatchNorm	1×1000×1×40	160
	1×1000×1×40	$x^{2}$-Activation	1×1000×1×40	-
	1×1000×1×40	Dropout (0.5)	1×1000×1×40	-
3	1×1000×1×40	Reshape	1000×40×1	-
	1000×40×1	AvgPool2d (75×1)	62×40×1	-
	62×40×1	log(x)-Activation	62×40×1	-
4	62×40×1	Flatten	2480	-
	6144	Softmax	*K*	4962
Total				10,922

## References

[1] Meng J., Zhang S., Bekyo A., Olsoe J., Baxter B., He B. (2016). Noninvasive Electroencephalogram Based Control of a Robotic Arm for Reach and Grasp Tasks. Sci. Rep..

[2] Carlson T., del R., Millan J. (2013). Brain-Controlled Wheelchairs: A Robotic Architecture. IEEE Robot. Autom. Mag..

[3] Lebedev M.A., Nicolelis M.A.L. (2017). Brain-Machine Interfaces: From Basic Science to Neuroprostheses and Neurorehabilitation. Physiol. Rev..

[4] Hochreiter S., Schmidhuber J. (1997). Long short-term memory. Neural Comput..

[5] Lecun Y., Bengio Y., Hinton G. (2015). Deep learning. Nature.

[6] Tabar Y.R., Halici U. (2016). A novel deep learning approach for classification of EEG motor imagery signals. J. Neural Eng..

[7] Thomas J., Maszczyk T., Sinha N., Kluge T., Dauwels J. Deep learning-based classification for brain-computer interfaces. Proceedings of the 2017 IEEE International Conference on Systems, Man, and Cybernetics (SMC).

[8] Sakhavi S., Guan C., Yan S. (2018). Learning Temporal Information for Brain-Computer Interface Using Convolutional Neural Networks. IEEE Trans. Neural Netw. Learn. Syst..

[9] Zhang J., Yan C., Gong X. Deep convolutional neural network for decoding motor imagery based brain computer interface. Proceedings of the 2017 IEEE International Conference on Signal Processing, Communications and Computing (ICSPCC).

[10] Leeb R., Brunner C., Mueller-Put G., Schloegl A., Pfurtscheller G. (2008). BCI Competition 2008-Graz Data Set b.

[11] Greaves A.S. Classification of EEG with Recurrent Neural Networks. https://cs224d.stanford.edu/reports/GreavesAlex.pdf.

[12] Forney E.M., Anderson C.W. Classification of EEG during imagined mental tasks by forecasting with Elman Recurrent Neural Networks. Proceedings of the The 2011 International Joint Conference on Neural Networks.

[13] Hema C.R., Paulraj M.P., Yaacob S., Adom A.H., Nagarajan R. Recognition of motor imagery of hand movements for a BMI using PCA features. Proceedings of the 2008 International Conference on Electronic Design.

[14] Zhang X., Yao L., Huang C., Sheng Q.Z., Wang X. (2017). Enhancing mind controlled smart living through recurrent neural networks. arXiv.

[15] Goldberger A.L., Amaral L., Glass L., Hausdorff J.M., Ivanov P.C., Mark R.G., Mietus J.E., Moody G., Peng C., Stanley H. (2000). PhysioBank, PhysioToolkit, and PhysioNet: Components of a New Research Resource for Complex Physiologic Signals. Circulation.

[16] An J., Cho S. Hand motion identification of grasp-and-lift task from electroencephalography recordings using recurrent neural networks. Proceedings of the 2016 International Conference on Big Data and Smart Computing (BigComp).

[17] Cho K., Merrienboer B.V., Bahdanau D., Bengio Y. (2014). On the properties of neural machine translation: Encoder-decoder approaches. arXiv.

[18] Schirrmeister R.T., Springenberg J.T., Fiederer L.D.J., Glasstetter M., Eggensperger K., Tangermann M., Hutter F., Burgard W., Ball T. (2017). Deep learning with convolutional neural networks for EEG decoding and visualization. Hum. Brain Mapp..

[19] Lawhern V.J., Solon A.J., Waytowich N.R., Gordon S.M., Hung C.P., Lance B.J. (2016). EEGnet: A compact convolutional network for EEG-based brain-computer interfaces. arXiv.

[20] Bashivan P., Rish I., Yeasin M., Codella N. (2015). Learning representations from EEG with deep recurrent-convolutional neural networks. arXiv.

[21] Popov E., Fomenkov S. Classification of hand motions in EEG signals using recurrent neural networks. Proceedings of the 2016 2nd International Conference on Industrial Engineering, Applications and Manufacturing (ICIEAM).

[22] (2017). Guger Technologies. http://www.gtec.at/.

[23] Tayeb Z., Waniek N., Fedjaev J., Ghaboosi N., Rychly L., Widderich C., Richter C., Braun J., Saveriano M., Cheng G. (2018). Gumpy: A Python toolbox suitable for hybrid brain–computer interfaces. J. Neural Eng..

[24] Nolan H., Whelan R., Reilly R. (2010). FASTER: Fully Automated Statistical Thresholding for EEG artifact Rejection. J. Neurosci. Methods.

[25] Team T.T.D. (2016). Theano: A Python framework for fast computation of mathematical expressions. arXiv.

[26] Chollet F. Keras, 2015. https://github.com/fchollet/keras.

[27] Erhan D., Bengio Y., Courville A., Manzagol P.A., Vincent P., Bengio S. (2010). Why Does Unsupervised Pre-training Help Deep Learning?. J. Mach. Learn..

[28] Sun D.L., Smith J.O. (2012). Estimating a Signal from a Magnitude Spectrogram via Convex Optimization. arXiv.

[29] Pfurtscheller G., da Silva F.H.L. (1999). Event-related EEG/MEG synchronization and desynchronization: Basic principles. Clin. Neurophysiol..

[30] loffe S., Szegedy C. (2015). Batch Normalization: Accelerating Deep Network Training by Reducing Internal Covariate Shift. arXiv.

[31] Ioffe S., Szegedy C. (2014). Adam: A method for stochastic optimization. arXiv.

[32] Liang M., Hu X. Recurrent convolutional neural network for object recognition. Proceedings of the 2015 IEEE Conference on Computer Vision and Pattern Recognition (CVPR).

[33] Grosse-Wentrup M., Buss M. (2008). Multiclass Common Spatial Patterns and Information Theoretic Feature Extraction. IEEE Trans. Biomed. Eng..

[34] Brodu N., Lotte F., Lecuyer A. Comparative study of band-power extraction techniques for Motor Imagery classification. Proceedings of the 2011 IEEE Symposium on Computational Intelligence, Cognitive Algorithms, Mind, and Brain (CCMB).

[35] Gerking J.M., Pfurtscheller G., Flyvbjerg H. (1999). Designing optimal spatial filters for single-trial EEG classification in a movement task. Clin. Neurophysiol..

[36] Sherwani F., Shanta S., Ibrahim B.S.K.K., Huq M.S. Wavelet based feature extraction for classification of motor imagery signals. Proceedings of the 2016 IEEE EMBS Conference on Biomedical Engineering and Sciences (IECBES).

[37] Pudil P., Novovicova J., Kittler J. (2008). Floating search methods in feature selection. Pattern Recognit. Lett..

[38] Lab Streaming Layer. https://github.com/sccn/labstreaminglayer.

[39] Hessel M., Soyer H., Espeholt L., Czarnecki W., Schmitt S., van Hasselt H. (2018). Multi-task deep reinforcement learning with popart. arXiv.

[40] Li Z., Wang Y., Zhi T., Chen T. (2017). A survey of neural network accelerators. Front. Comput. Sci..

[41] Merolla P.V., Arthur J.V., Alvarez-Icaza R., Cassidy A.S., Sawada J., Akopyan F., Jackson B.L., Imam N., Guo C., Nakamura Y. (2014). A million spiking-neuron integrated circuit with a scalable communication network and interface. Science.

